# Exogenous Gonadotrophin Stimulation Induces Partial Maturation of Human Sertoli Cells in a Testicular Xenotransplantation Model for Fertility Preservation

**DOI:** 10.3390/jcm9010266

**Published:** 2020-01-18

**Authors:** Marsida Hutka, Lee B. Smith, Ellen Goossens, W. Hamish B. Wallace, Jan-Bernd Stukenborg, Rod T. Mitchell

**Affiliations:** 1 Medical Research Council (MRC) Centre for Reproductive Health, The University of Edinburgh, The Queen’s Medical Research Institute, 47 Little France Crescent, Edinburgh EH16 4TJ, UK; marsida.hutka@gmail.com (M.H.); lee.smith@ed.ac.uk (L.B.S.); 2 School of Environmental and Life Sciences, University of Newcastle, Callaghan 2308, Australia; 3 Biology of the Testis, Research Laboratory for Reproduction, Genetics and Regenerative Medicine, Vrije Universiteit Brussel (VUB), Laarbeeklaan 103, 1090 Brussels, Belgium; ellen.goossens@vub.be; 4 Department of Oncology and Haematology, Royal Hospital for Sick Children, 9 Sciennes Road, Edinburgh EH9 1LF, UK; hamish.wallace@nhs.net; 5 NORDFERTIL Research Lab Stockholm, Childhood Cancer Research Unit, Department of Women’s and Children’s Health, Karolinska Institutet and Karolinska University Hospital, Solna SE-17164, Sweden; jan-bernd.stukenborg@ki.se; 6 Department of Diabetes and Endocrinology, Royal Hospital for Sick Children, 9 Sciennes Road, Edinburgh EH9 1LF, UK

**Keywords:** Sertoli cell, Leydig cell, steroidogenesis, hCG, human fetal testis, xenotransplantation, fertility preservation, oncofertility

## Abstract

The future fertility of prepubertal boys with cancer may be irreversibly compromised by chemotherapy and/or radiotherapy. Successful spermatogenesis has not been achieved following the xenotransplantation of prepubertal human testis tissue, which is likely due to the failure of somatic cell maturation and function. We used a validated xenograft model to identify the factors required for Leydig and Sertoli cell development and function in immature human testis. Importantly, we compared the maturation status of Sertoli cells in xenografts with that of human testis tissues (*n* = 9, 1 year-adult). Human fetal testis (*n* = 6; 14–21 gestational weeks) tissue, which models many aspects of prepubertal testicular development, was transplanted subcutaneously into castrated immunocompromised mice for ~12 months. The mice received exogenous human chorionic gonadotropin (hCG; 20IU, 3×/week). In xenografts exposed continuously to hCG, we demonstrate the maintenance of Leydig cell steroidogenesis, the acquisition of features of Sertoli cell maturation (androgen receptor, lumen development), and the formation of the blood–testis barrier (connexin 43), none of which were present prior to the transplantation or in xenografts in which hCG was withdrawn after 7 months. These studies provide evidence that hCG plays a role in Sertoli cell maturation, which is relevant for future investigations, helping them generate functional gametes from immature testis tissue for clinical application.

## 1. Introduction

Survival rates for childhood cancer have increased over recent decades as a result of advancements in detection and treatment [1]. However, many patients receiving chemotherapy and/or radiotherapy for cancer or non-malignant conditions (e.g., blood disorders) encounter a broad spectrum of long-term side effects, including infertility [2,3,4,5]. The testes of prepubertal boys are composed of immature somatic and germ cell populations and do not begin to produce sperm until puberty. Therefore, these patients cannot benefit from the standard technologies of sperm freezing and there are currently no established clinical options to preserve their future fertility. For young boys, it may be possible to cryopreserve the immature testicular tissue and/or spermatogonial stem cells (SSCs) prior to gonadotoxic treatment for future clinical use [3]. A recent study involving non-human primate testis has provided proof of principle that the autotransplantation of prepubertal frozen-thawed testicular tissue might be a potential future fertility preservation strategy [6]. However, this approach has not been replicated in human studies and may not be safe for patients with haematological malignancies due to the risk of reintroducing malignant cells [7,8,9,10,11]. For these boys, subsequent fertility may require ex-situ differentiation of the immature germ cells present in prepubertal testis tissue into sperm, the latter can be used to generate offspring.

Sertoli cells are the only somatic cells located within the seminiferous tubules that are in close physical contact with undifferentiated germ cells. At puberty, mature Sertoli cells form an essential unique microenvironment through the development of the blood–testis barrier, which, among others, includes the expression of Connexin 43 (CX43, gap junction protein) and Claudin 11 (CLDN11, tight junction protein), restricted to the basement membrane. Under normal physiological circumstances, germ cell development begins at puberty and is dependent upon the establishment of a fully mature Sertoli cell population and testosterone production from Leydig cells [12,13]. Several lines of evidence point to the essential role of Sertoli cell maturation in supporting germ cell differentiation, including knockout mouse models in which the loss of function of genes expressed in mature Sertoli cells results in infertility [14,15,16]. In addition, it has been reported that the failure of Sertoli cells to attain a mature phenotype in humans may lead to impaired germ cell development [17].

Xenotransplantation is a system that has been used to study testis development in a variety of species [18]. This includes the xenografting of human fetal testis tissue, which shares many features with the prepubertal human testis, such as the absence of a lumen within the seminiferous cords, the presence of immature Sertoli cells, and undifferentiated germ cells [19,20].

In humans, Sertoli cells do not reach full maturity until puberty and the precise mechanisms for their maturation have not yet been determined. Previous studies involving the short-term xenografting of human fetal testis have demonstrated the limited development of the seminiferous cords [19,21,22]. Sertoli cell maturation and germ cell development have been shown when immature non-human primate testis grafts were exposed to hCG alone [23]. Therefore, we aimed to determine the role of hCG in Sertoli and germ cell maturation in the immature human testis. In this study, second-trimester human fetal testis fragments were transplanted subcutaneously into nude castrate mice for ~12 months, and host mice were exposed to hCG. We hypothesised that long-term, continuous hCG stimulation would maintain the steroidogenic function of the Leydig cells and induce Sertoli cell maturation in immature human testis xenografts.

## 2. Materials and Methods

### 2.1. Ethics Statement

The South East Scotland Research Ethics Committee approved the collection and use of human fetal (LREC08/S1101/1), prepubertal (LREC13/SS0145), and adult (LREC10/S1402/33) testicular tissues. Ethical approval for the receipt and use of pre(peri)pubertal human testis tissue was also obtained from the internal review board of the UZ Brussel (2014/328). Patients and parents agreed to donate testis tissue for research and signed an informed consent.

### 2.2. Human Testis Tissue Collection

Second-trimester human fetal testes (*n* = 6, 14–21 gestational weeks (GW)) were obtained following elective terminations of pregnancy. None of the terminations were for reasons of fetal abnormalities. Small fragments of testis tissue were fixed for 2 h in Bouin’s fixative (Clin-Tech Guilford, Guildford, UK) as pre-graft controls. The remainder of the tissue was placed in Liebowitz L-15 media supplemented with glutamine, 10% fetal bovine serum, 1% penicillin/streptomycin, 1% nonessential amino acids (all Sigma–Aldrich, Poole, UK), and was subsequently used for xenograft studies. Pre(peri)pubertal human testes (*n* = 6, aged 1–13 years) were collected from boys with cancer or non-malignant diseases undergoing testicular biopsy for fertility preservation purposes. A small portion of each biopsy was available and the remaining piece was cryopreserved for future clinical application. Adult human testes (*n* = 3), with full spermatogenesis, were collected from patients undergoing orchiectomy.

### 2.3. Xenotransplantation Procedure

Animal studies were performed in accordance with the UK Home Office Animals (Scientific Procedures) Act 1986 under the project licence PPL 60/4564. Animals were housed in individually ventilated cages (IVCs; Tecniplast, Varese, Italy) in a room with fixed lighting from 07:00–19:00. The temperature was maintained at 20–25 °C and the humidity was kept at ~55%. Drinking water and soy-free chow were available ad libitum. Animals were acclimatised for at least one week prior to entry into experiments.

Adult male CD1 nude mice were anaesthetized by inhalation of isoflurane. To eliminate endogenous testosterone production in the host mice, castration was performed through a longitudinal scrotal incision. Human fetal testes (*n* = 6; 14–21 GW) were cut into 86 fragments (~1 mm^3^) and for each fetus 3–6 fragments were inserted subcutaneously under the dorsal skin of the host nude mice (*n* = 21, aged 4–6 weeks, Charles River, UK) using a 13 G cancer implant needle (Popper and Sons, New York, NY, USA) as previously described [19]. During the first 5 days post-surgery, the analgesic Carprofen (Rimadyl; Pfizer, New York, NY, USA) and the antibiotic Baytril (Enrofloxacin; Bayer, Leverkusen, Germany) were added to the drinking water.

### 2.4. Treatment Regimens

For each experiment, host mice received the tissue from one fetus and were randomly allocated to one of three groups: ‘Untreated’, ‘Continuous hCG’, and ‘Withdrawal hCG’. ‘Untreated’ mice (*n* = 12; 4 died prematurely) did not receive any treatment for 9–12 months; whilst mice belonging to the ‘Continuous hCG’ group (*n* = 7) received exogenous hCG for 9–12 months. In the ‘Withdrawal hCG’ group, mice (*n* = 2) were exposed to hCG for 7 months (to mimic fetal and infancy periods during which gonadotrophins are high), followed by 5 months without hCG (to mimic prepuberty in which gonadotrophin levels are low/undetectable). For hCG (20 IU/0.1 mL; Ovitrelle, Merck Serono, Feltham, UK) treatment, mice were injected subcutaneously three times a week commencing one week after grafting.

### 2.5. Retrieval of Testicular Xenografts

Host mice were culled via the inhalation of carbon dioxide (CO_2_) followed by cervical dislocation. The body weight of nude mice was recorded; the seminal vesicle weight of each host mouse was also reported as a biomarker of testosterone production by grafts. Grafts were visually identified as masses located on the interior surface of the dorsal skin, excised from the skin and fixed for 2–3 h in Bouin’s solution.

### 2.6. Histological Analysis

After fixation, testis tissues were immersed overnight in a series of graded alcohols (70–100%) in an automated Leica tissue processor (Leica Microsystem, Milton Keynes, UK). Subsequently, tissue fragments were placed into blocks of molten paraffin wax by the histology team (Shared University Research Facilities (SuRF), Edinburgh). Blocks were cooled on ice prior to sectioning. A Leica RM2135 microtome (Leica) was used to cut paraffin blocks into 5 μm serial sections, which were placed in a heated water bath (Lamb RA, model E/65) at 45 °C to avoid any creases in the section. Individual sections were mounted onto electrostatically charged pre-labelled slides (Leica Biosystems Peterborough Limited, Berkshire, UK). Slides were dried overnight in an incubator (Lamb RA, model E28.5) at 55 °C.

All grafts were sectioned and immunostained. To ensure reproducibility, at least 2–3 Sections per graft (*n* = 31 grafts retrieved) were assessed and a panel of 15 proteins was examined. Sections were placed in xylene to remove paraffin and rehydrated by immersion in decreasing concentrations of ethanol. Subsequently, sections were submerged in haematoxylin and eosin to stain nuclei and cytoplasm, respectively. Next, sections were dehydrated through increasing concentrations of ethanol and then placed in xylene. Sections were then mounted with glass coverslips (VWR International, Lutterworth, UK) using DPX mountant (Sigma-Aldrich, Dorset, UK). Images were examined using a Provis microscope (Olympus Optical, London, UK) and were captured using an attached digital camera (Canon DS126161, Surrey, UK).

### 2.7. Immunofluorescence and Confocal Microscopic Analysis

For the fluorescent immunostaining, slides were dewaxed and heat-induced epitope retrieval was performed in 0.01 M citrate buffer (pH 6) using a Decloacking Chamber^TM^ Pro (Biocare Medical, Pacheco, CA, USA). Endogenous peroxidase activity was quenched by the immersion of sections in hydrogen peroxide and methanol. This was followed by a blocking serum step. Slides were then incubated overnight in a humidified chamber with primary antibodies diluted in blocking serum solution. Negative controls included the omission of the primary antibody. The following day, slides were incubated with HRP-conjugated secondary antibodies (Santa Cruz Biotechnology, Heidelberg, Germany), dye-labelled tyramide (TSA; Perkin Elmer, Waltham, MA, USA), counterstained with Hoechst (Thermo Fisher Scientific, Waltham, MA, USA) and then mounted with PermaFluor (Thermo Fisher Scientific). The antibodies used for immunofluorescence are listed in Table 1. Images were acquired using an LSM 780 confocal microscope (Carl Zeiss, Cambridge, UK) with Zen imaging software LSM780 (Carl Zeiss, Cambridge, UK).

### 2.8. Statistical Analysis

A statistical analysis was carried out on GraphPad Prism 7 (La Jolla, CA, USA). The differences between two groups were analysed using an unpaired *t*-test. The results are presented as a mean ± SD. *p* < 0.05 was considered statistically significant.

## 3. Results

### 3.1. Human Fetal Testis Xenograft Recovery

**Figure 1:**
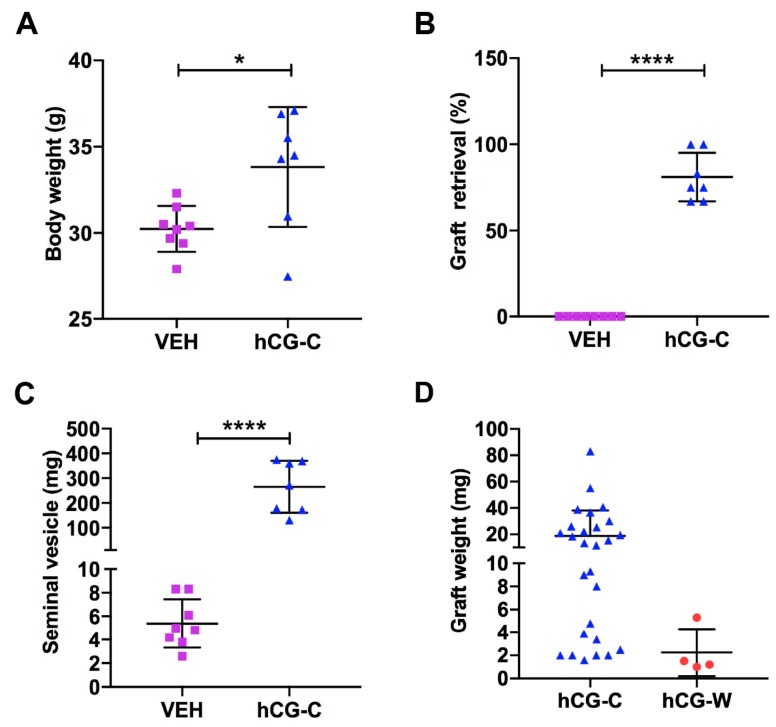
(**A**) Body weight of host mice from ‘Untreated’ vehicle (VEH, *n* = 8) and ‘Continuous hCG’ (hCG-C, *n* = 7) groups. (**B**) Graft retrieval rate from individual host mice in ‘Untreated’ (*n* = 8) and ‘Continuous hCG’ (*n* = 7) groups. (**C**) Seminal vesicle weight of host mice from ‘Untreated’ (*n* = 8) and ‘Continuous hCG’ (*n* = 7) groups. (**D**) Graft weights from ‘Continuous hCG’ (hCG-C, *n* = 27) and ‘Withdrawal hCG’ groups (hCG-W, *n* = 4). * *p* < 0.05, **** *p* < 0.0001.

Host mice in the ‘Continuous hCG’ group had a significantly higher mean body weight compared with ‘Untreated’ vehicle controls (33.8 vs. 30.2 g, *p* < 0.05; Figure 1A).

For ‘Continuous hCG’ and ‘Untreated’ controls, a total of 78 fragments (~1 mm^3^) of second-trimester human fetal testes (*n* = 5; 14–21 GW) were grafted into castrate immunodeficient mice (*n* = 19) for 9–12 months. No grafts (0/44) were retrieved from the ‘Untreated’ group, whilst 79% of the grafts (27/34) survived in the ‘Continuous hCG’ group (Figure 1B). In mice exposed to ‘Continuous hCG’, the mean seminal vesicle weight was significantly higher than that of the ‘Untreated’ group (265.5 ± 39.6 vs. 5.4 ± 0.7; *p* < 0.0001; Figure 1C), which is consistent with the testosterone production of the ‘Continuous hCG’ group and the lack of testosterone biosynthesis in the ‘Untreated’ group.

Given that no grafts survived in the ‘Untreated’ group, we included a ‘Withdrawal hCG’ group in which 50% of the grafts (4/8) were recovered. Continuously hCG-treated grafts showed an increase in graft weight compared to ‘Withdrawal hCG’ (Figure 1D). Overall, the mean graft weight was higher in ‘Continuous hCG’ (*n* = 27) compared to the ‘Withdrawal hCG’ group (*n* = 4) (18.8 ± 3.7 vs. 2.3 ± 1.0; *p* > 0.05; Figure 1D), although this did not achieve statistical significance.

These results suggest that hCG in the initial phase is required to support the survival of human fetal testis xenografts. For the remainder of the study, we focused on a comparison of testicular xenograft development between ‘Continuous hCG’ and ‘Withdrawal hCG’.

### 3.2. Lumen Development in Long-Term Continuously hCG-Exposed Testis Xenografts

**Figure 2:**
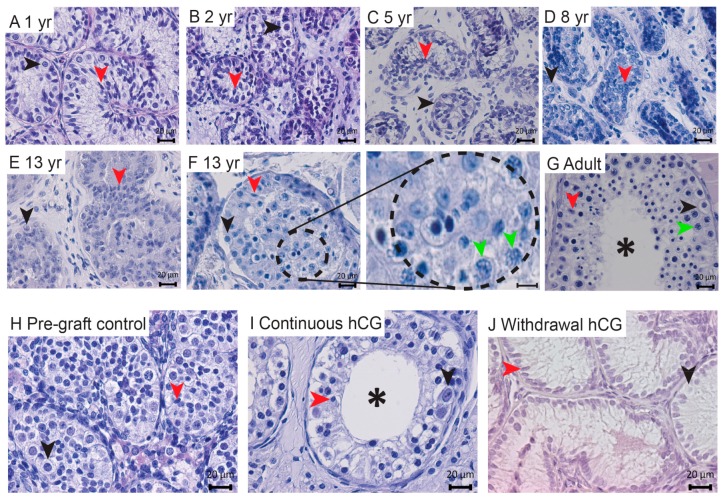
Continuous hCG stimulation induces lumen development in long-term human fetal testis xenografts. (**A**–**G**) The histological evaluation of human testis tissue architecture across development. (**F**) The right panel shows a higher magnification of the encircled area in the left panel. (**H**,**J**) No signs of lumen development were observed in any of the pre-graft control tissues (14 GW) or the withdrawal hCG group (7 months hCG followed by 5 months withdrawal hCG). (**I**) Continuous hCG exposure induced lumen formation in all long-term xenografts (9 months). The images in (**I**,**J**) are representative of *n* = 27 and *n* = 4, respectively. The asterisks depict seminiferous tubules with a developed lumen. Red arrowheads denote Sertoli cells. The black arrowheads indicate immature germ cells (spermatogonia). The green arrowheads show meiotic cells (spermatocytes). yr: years. Scale bar: 20 μm.

In order to determine the normal development of human testicular tissues from childhood to adulthood, we assessed the development of pre(peri)pubertal (*n* = 6; aged 1–13 years) and adult (*n* = 3) human testis control tissues. When examined histologically, seminiferous cords/tubules containing Sertoli and germ cells surrounded by the interstitial compartment were identified in ungrafted testis tissues (Figure 2A–G). Meiotic cells were detected in peripubertal and adult patients (Figure 2F,G). Lumen development was not observed in pre(peri)pubertal boys (Figure 2A–F), whilst the presence of lumen was evident within the seminiferous tubules of adult testes (Figure 2G).

A histological analysis of the grafts revealed somatic and germ cell survival (Figure 2I,J). Seminiferous cords, with a developed lumen, were seen in all (27/27) continuously hCG-treated grafts (Figure 2I), although occasional seminiferous cords displayed incomplete lumen development. No signs of lumen formation were observed in any (0/4) of the sections from respective pre-graft controls and the ‘Withdrawal hCG’ group (Figure 2H,J).

### 3.3. Steroidogenesis in Long-Term Continuously hCG-Exposed Testis Xenografts

**Figure 3:**
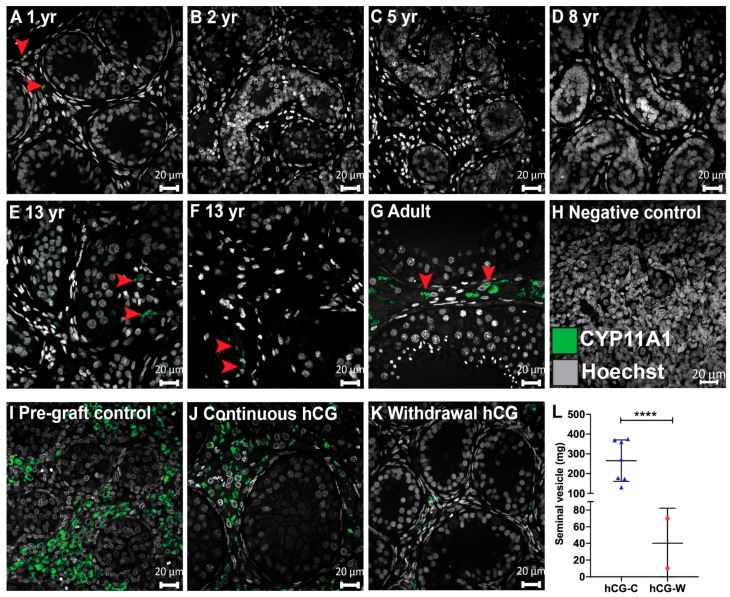
Maintenance of steroidogenesis in long-term continuously hCG-exposed human fetal testis xenografts. (**A**–**G)** The characterisation of CYP11A1 (steroidogenic marker) expression across human testis development. (**H**) Negative control (second-trimester human fetal testis). (**I**,**J**) Expression of CYP11A1 was observed in all pre-graft control testis tissues (17 GW) and maintained in continuously hCG-treated xenografts (9 months hCG). (**K**) Occasional CYP11A1^+^ cells were observed in ‘Withdrawal hCG’ grafts (7 months hCG followed by 5 months withdrawal hCG). Images in (**J**,**K**) are representative of *n* = 27 and *n* = 4, respectively. (**L**) The seminal vesicle weight of castrate host mice from the ‘Continuous hCG’ (*n* = 7) and ‘Withdrawal hCG’ groups (*n* = 2). **** *p* < 0.0001. The arrowheads point to CYP11A1^+^ cells. yr: years. Scale bar: 20 μm.

Castration results in the cessation of endogenous testosterone production from the host mice. To assess the responsiveness of Leydig cells within xenografts to exogenous hCG, we analysed the expression of cholesterol side-chain cleavage (CYP11A1), which is an enzyme that catalyses the first and rate-limiting step of steroidogenesis. First, we characterized CYP11A1 expression in ungrafted human testis tissues (1 yr-adult) (Figure 3A–G). CYP11A1-expressing cells were rare in the testis of the 1-year-old boy (Figure 3A), whereas no CYP11A1 expression was identified in prepuberty between 2 and 8 years of age (Figure 3B–D). Thereafter, occasional CYP11A1^+^ cells were observed in both 13-year-old boys (Figure 3E,F). As expected, CYP11A1 expression was demonstrated in adult testis tissue (Figure 3G). An examination of the fetal tissues used for xenografting revealed CYP11A1 in the interstitial compartment of pre-graft controls with maintenance of expression in all continuously hCG-exposed grafts (Figure 3I,J). In contrast, CYP11A1^+^ cells were very rarely seen in the ‘Withdrawal hCG’ group (Figure 3K).

Moreover, the seminal vesicles of host mice were dissected out and their weight was recorded as an indirect indicator of testosterone production by testicular xenografts. Seminal vesicle weights were significantly higher in the ‘Continuous hCG’ group compared with the ‘Withdrawal hCG’ group (265.5 ± 39.6 vs. 40.1 ± 29.9; *p* < 0.0001, Figure 3L). Seminal vesicle weights in mice continuously exposed to hCG were similar to the pre-castration adult mouse values, indicating that the xenografts are producing testosterone [24].

Taken together, these results indicate that continuous hCG is required to maintain steroidogenesis in fetal human testis.

### 3.4. Expression of Immature Sertoli Cell Markers in Testis Xenografts

To evaluate the status of Sertoli cell maturation in grafts, we analyzed a panel of immunohistochemical markers. We used Cytokeratin 18 (CK18), a type I intermediate filament protein to identify immature (CK18^+^) Sertoli cells and Ki67, a cell proliferation marker, to identify mature (Ki67^−^) Sertoli cells.

**Figure 4:**
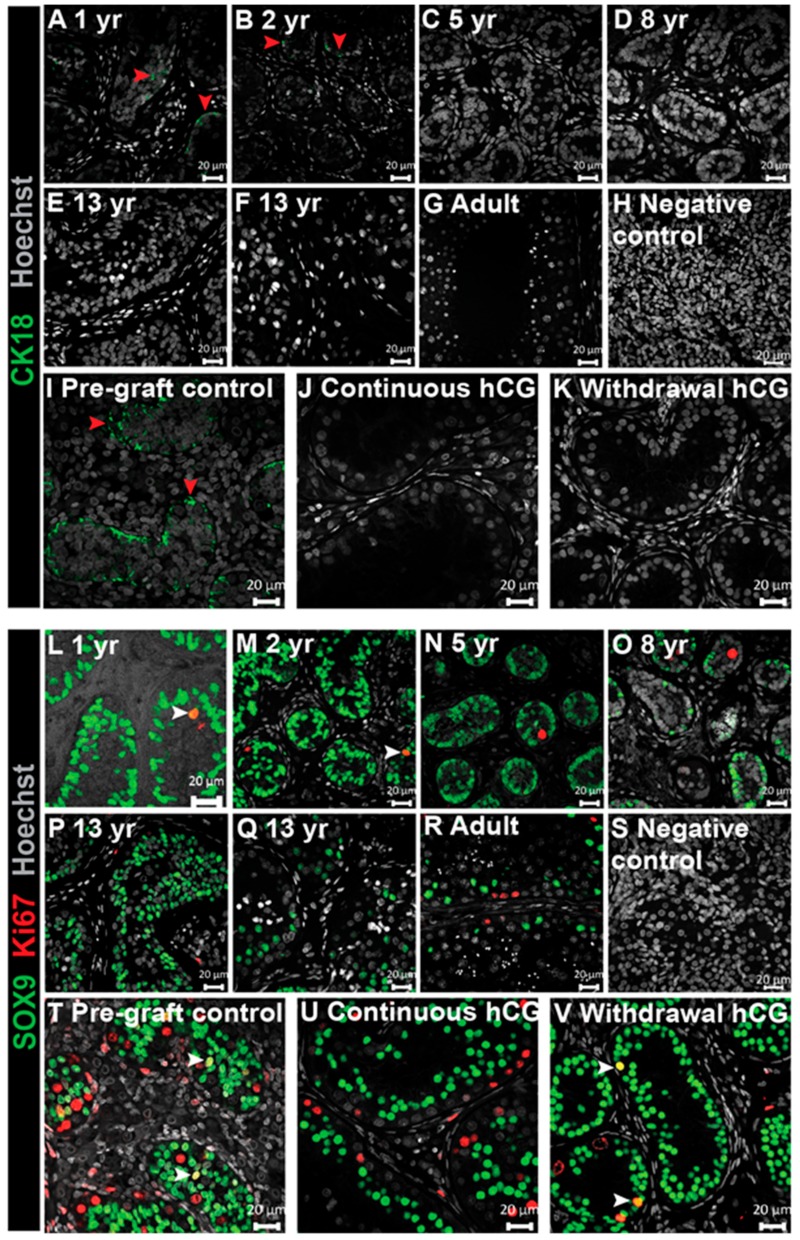
Downregulation of immature Sertoli cell features in long-term continuously hCG-exposed human fetal testis xenografts. (**A**–**G**) The characterisation of CK18 (Sertoli cell marker) expression across human testis development. (**H**) The negative control (second-trimester human fetal testis). (**I**–**K**) Immunopositive staining for CK18 was found in all pre-graft control testis tissues (17 GW) but not in long-term xenografts exposed continuously to hCG (12 months hCG) or ‘Withdrawal hCG’ grafts (7 months hCG followed by 5 months withdrawal hCG). The images in (**J**,**K**) are representative of *n* = 27 and *n* = 4, respectively. The red arrowheads denote CK18^+^ cells. (**L**–**R**) The characterisation of Sertoli cell proliferation across human testis development. (**S**) Negative control (second-trimester human fetal testis). (**T**–**V**) Proliferating Sertoli cells were found in the pre-graft control and withdrawal hCG group (7 months hCG followed by 5 months withdrawal hCG) in comparison to SOX9^+^/Ki67^−^ seminiferous cords in continuously hCG-exposed xenografts (12 months hCG). The images in (**U**) and (**V**) are representative of *n* = 27 and *n* = 4, respectively. The white arrowheads identify SOX9^+^/Ki67^+^ cells. yr: years. Scale bar: 20 μm.

First, we determined the pattern of CK18^+^ expression in human testis tissue from early childhood through to adulthood. CK18^+^ cells were seen in Sertoli cells at the youngest ages (1–2 years; Figure 4A,B), whilst no expression was detected in human testis by the age of 5 years onwards, including adult human testis (Figure 4C–G).

We also investigated CK18 expression in human fetal testis xenografts. Pre-graft controls expressed CK18, indicating the immaturity of the Sertoli cells, whilst none of the grafts taken from either group, ‘Continuous hCG’ or ‘Withdrawal hCG’, expressed CK18 (Figure 4I–K), suggesting that the Sertoli cells have commenced early maturation.

Immature Sertoli cells are reported to undergo proliferation during neonatal and peripubertal periods and to cease proliferation in adulthood, with the final Sertoli cell number determining sperm output in adulthood [25]. We determined the presence of Sertoli cell proliferation in human testis tissue from early childhood through to adulthood. Sertoli cells were identified by expression of SRY-Box 9 (SOX9), whilst proliferative cells were detected by expression of Ki67. Mitotic Sertoli cells (SOX9^+^/Ki67^+^) were found within the testes of the youngest boys (1–2 years) (Figure 4L,M), whereas Sertoli cells were non-proliferative in (peri)pubertal and adult testes (Figure 4N–R). Interestingly, we did not identify proliferative Sertoli cells in the two (peri)pubertal patients.

For fetal xenografts, proliferative Sertoli cells (SOX9^+^/Ki67^+^) were present in pre-graft controls, consistent with their immature status (Figure 4T). In all (27/27) continuously hCG-treated grafts, Sertoli cell proliferation had ceased (Figure 4U); however, Sertoli cell proliferation was maintained in 4/4 xenografts from the ‘Withdrawal hCG’ group (Figure 4T,V and Figure S1). Taken together, these results suggest that the cessation of Sertoli cell proliferation occurs with the maturation of Sertoli cells in the human testis and that this can be promoted by continuous exposure to hCG in human fetal testis xenografts.

### 3.5. Analysis of Junctional Proteins in Testis Xenografts

At puberty, inter-Sertoli cell junctions form the blood–testis barrier (BTB), which protects differentiating germ cells from the immune system. CX43 and CLDN11 are reported to be key components of the BTB in rodent [16,26,27,28,29].

**Figure 5:**
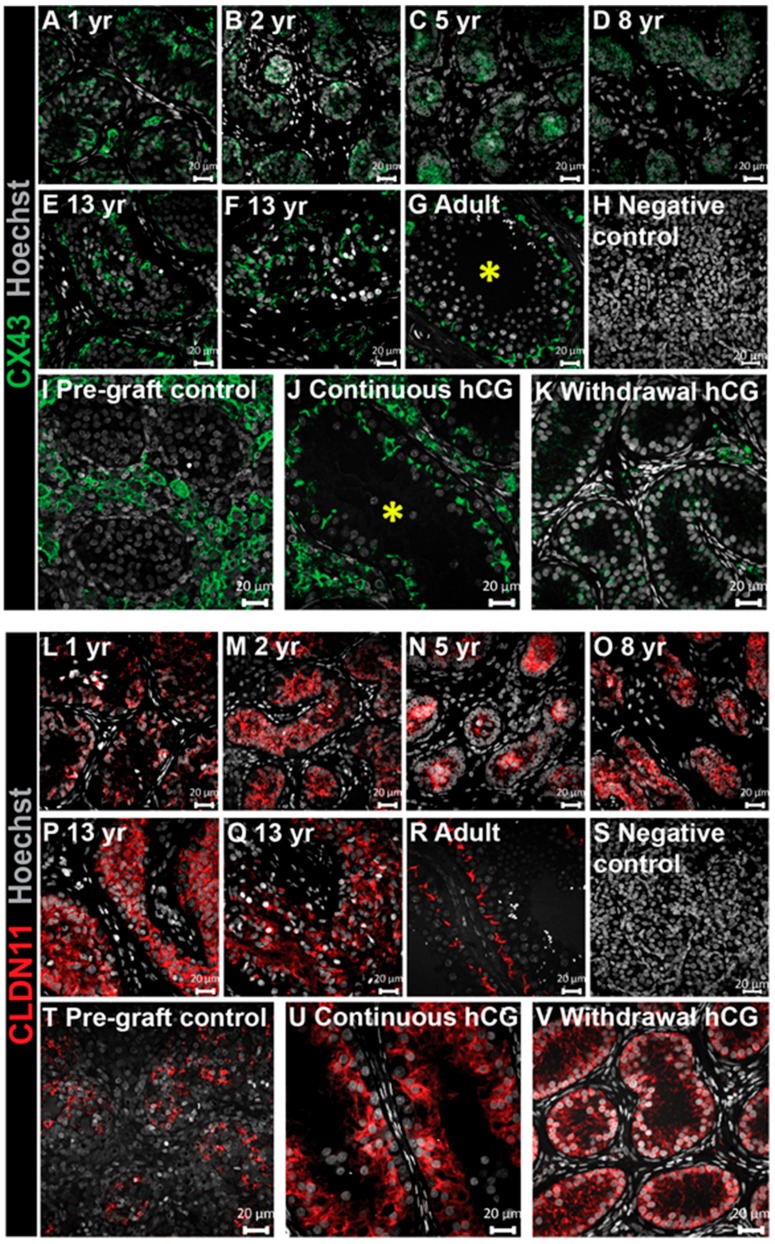
Continuous hCG stimulation promotes the appearance of a mature CX43 expression profile and maintains an immature CLDN11 distribution pattern in long-term human fetal testis xenografts. (**A**–**G**) CX43 expression across human testis development. (**H**) The negative control (second-trimester human fetal testis). (**I**) CX43 was absent within the seminiferous cords of the pre-graft control testis tissue (17 GW). (**J**) Continuous hCG exposure induced the expression of a mature CX43 distribution profile in long-term xenografts (12 months hCG). (**K**) Faint CX43 expression was observed in the ‘Withdrawal hCG’ group (7 months hCG followed by 5 months withdrawal hCG). Images in (**J**,**K**) are representative of *n* = 27 and *n* = 4, respectively. The asterisks depict seminiferous tubules with a mature CX43 profile. (**L**–**R**) CLDN11 expression (Sertoli cell marker) across human testis development. (**S**) The negative control (second-trimester human fetal testis). (**T**–**V**) Seminiferous cords in the pre-graft control (14 GW) and long-term xenografts exposed continuously to hCG (12 months hCG) and ‘Withdrawal hCG’ grafts (7 months hCG followed by 5 months withdrawal hCG) exhibited an immature CLDN11 profile. Images in (**U**,**V**) are representative of *n* = 27 and *n* = 4, respectively. yr: years. Scale bar: 20 μm.

We determined organizational patterns in human testis tissues across childhood and into adulthood. We identified a progressive change in CX43 distribution with increasing age. In the youngest patients, diffuse staining within the seminiferous cords was identified (Figure 5A–D), whilst localization was restricted to areas adjacent to the basement membrane and the centre of the tubules in both (peri)pubertal (13-year-old) boys (Figure 5E,F). A similar filamentous CX43 pattern between adjacent basal cells was noted in adult men (Figure 5G).

For xenografts, CX43 expression was exclusively located in the interstitial compartment with no staining found within the seminiferous cords of all pre-graft controls (Figure 5I). The xenografts exposed continuously to hCG had all (27/27) begun to express CX43 in the Sertoli cells adjacent to the basement membrane (Figure 5J), similar to that seen in adult testes (Figure 5G). In the ‘Withdrawal hCG’ group, faint CX43 staining was seen within the seminiferous cords and interstitial space in all (4/4) xenografts (Figure 5K).

We also investigated CLDN11 expression in ungrafted human testis tissues and human testis xenografts. We observed a developmental stage-specific switch from diffuse staining in seminiferous cords during childhood (Figure 5L–Q) to a mature expression profile restricted to the basal compartment in adult testes (Figure 5R). Interestingly, in (peri)pubertal human testis tissues, CLDN11 retained an ‘immature’ diffuse staining pattern.

Pre-graft controls exhibited an immature diffuse CLDN11 distribution and this pattern was retained in all xenografts, regardless of whether they were exposed to ‘Continuous hCG’ or ‘Withdrawal hCG’. (Figure 5T–V). Taken together, these results show a progressive development of the BTB from (peri)puberty (CX43 localisation) into adulthood (CLDN11 localisation) and that continuous exposure to hCG promotes the initial development of the BTB (CX43 localisation).

### 3.6. Expression of AMH and AR in Testis Xenografts

To gain further insight into the status of Sertoli cell maturation within grafts, double immunofluorescence was undertaken for the anti-Müllerian hormone (AMH), a marker reported to represent immature Sertoli cells, and the androgen receptor (AR), an indicator of Sertoli cell maturation [29].

**Figure 6:**
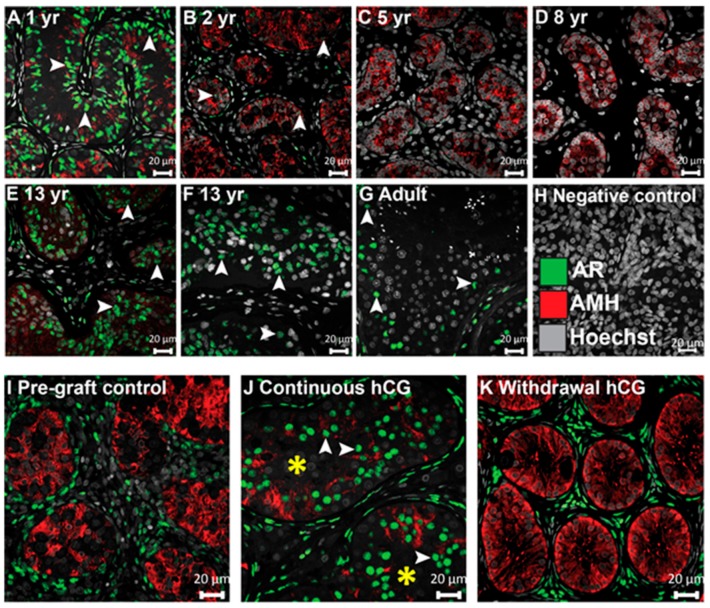
Continuous hCG stimulation induces AR expression in Sertoli cells but fails to suppress AMH expression in long-term human fetal testis xenografts. (**A**–**G**) Characterisation of AR and AMH expression across human testis development. (**H**) Negative control (second-trimester human fetal testis). (**I**–**K**) Continuous hCG exposure induced AR expression in Sertoli cells (9 months hCG) and maintained AMH in comparison to respective pre-graft control testis tissue (17 GW) and ‘Withdrawal hCG’ group (7 months hCG followed by 5 months withdrawal hCG) which displayed AR^−^/AMH^+^ Sertoli cells. White arrowheads point to AR^+^ Sertoli cells. Images in (**J**,**K**) are representative of *n* = 27 and *n* = 4, respectively. Asterisks depict AR^+^ seminiferous tubules, which were absent prior to transplantation (pre-graft control) and ‘Withdrawal hCG’ grafts. yr: years. Scale bar: 20 μm.

First, we determined the expression of the AMH and AR in human testis from infancy to adulthood. The AMH was expressed within the seminiferous cords of the 15-month-old patient (Figure 6A). AMH expression was maintained for the remainder of prepuberty with a gradual loss with advancing age (Figure 6B–G). The AR was expressed in a large proportion of Sertoli cells in the testis of the 1-year-old boy but was rarely expressed in the seminiferous cords of the 2-year-old boy (Figure 6B). Subsequently, the AR was not expressed in the Sertoli cells during mid-childhood (5–8 years of age) (Figure 6C,D). Thereafter, AR expression was identified in the Sertoli cells of the testes from the (peri)pubertal (13-year-old) and adult patients (Figure 6E–G).

For tissues used in xenograft experiments, there was no AR expression in the Sertoli cells of any of the pre-graft control testis tissues, whereas the majority of Sertoli cells acquired AR expression in all (27/27) grafts exposed continuously to hCG (Figure 6I,J). In grafts recovered from the ‘Withdrawal hCG’ group, the AR in Sertoli cells was not detected in any (0/4) of the grafts (Figure 6K). These results indicate that AR (re)expression in Sertoli cells is induced around puberty and can be promoted in human testis xenografts by hCG stimulation. The persistence of AMH in hCG-exposed xenografts indicates a partial maturation of Sertoli cells.

### 3.7. Assessment of Germ Cell Survival and Differentiation in Long-Term Testis Xenografts

**Figure 7:**
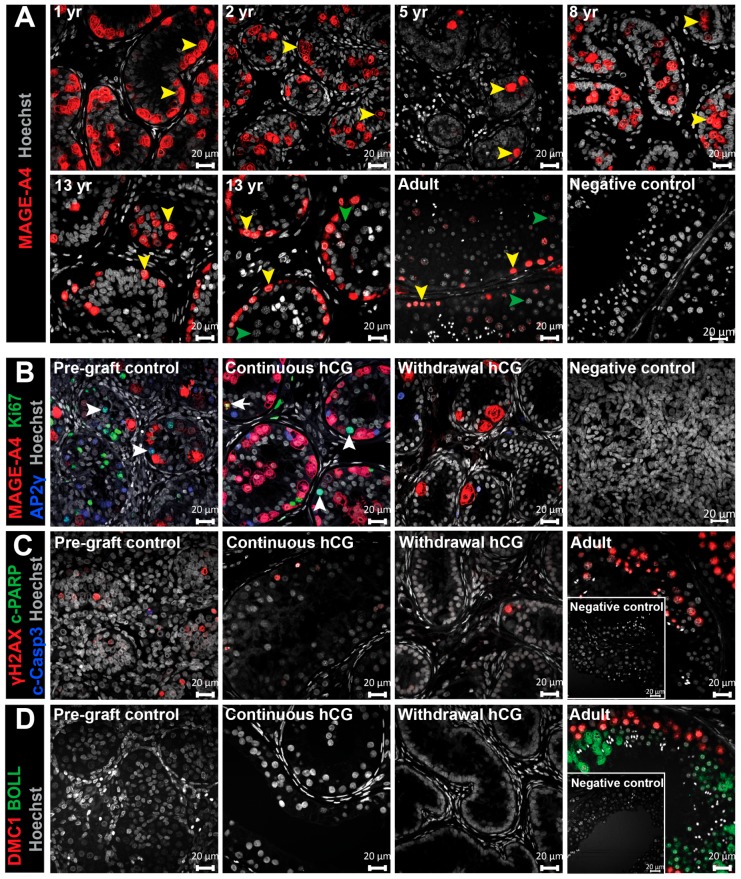
Continuous hCG stimulation supports germ cell survival but does not promote germ cell differentiation in long-term human fetal testis xenografts. (**A**) The distribution of MAGE-A4^+^ cells (spermatogonia marker) across human testis development. The negative control (adult human testis). The yellow arrowheads indicate spermatogonia. The green arrowheads point to meiotic cells. (**B**) Immunofluorescent analysis of MAGE-A4 (spermatogonia marker), AP2γ (gonocyte marker), and Ki67 (proliferation marker) expression. Spermatogonia and gonocytes were observed in both the pre-graft control testis tissue and in continuously hCG-treated xenografts (12 months hCG) and the ‘Withdrawal hCG’ group (7 months hCG followed by 5 months withdrawal hCG). The negative control (second-trimester human fetal testis). The white arrowheads denote AP2γ^+^/Ki67^+^ cells. The arrow points to a proliferating MAGE-A4^+^/Ki67^+^ cell. (**C**) Immunofluorescent analysis of γH2AX (meiotic- and apoptotic marker), c-PARP (apoptotic marker), and c-Casp3 (apoptotic marker) expression. Occasional γH2AX^+^ cells, which were c-PARP^−^/c-Casp3^−^, are found within the seminiferous cords of the pre-graft control and in xenografts from the ‘Continuous hCG’ (12 months hCG) and ‘Withdrawal hCG’ (7 months hCG followed by 5 months withdrawal hCG) groups. Adult testis tissue shows γH2AX^+^ meiotic and post-meiotic cells within the seminiferous tubule. The Negative control (adult human testis). (**D**) Immunofluorescent analysis of DNA meiotic recombinase 1 (DMC1) (meiotic marker) and boule-like protein (BOLL) (meiotic marker) expression. The pre-graft control testis tissue (14 GW) and xenografts from ‘Continuous hCG’ (9 months hCG) and ‘Withdrawal hCG’ (7 months hCG followed by 5 months withdrawal hCG) were negative for DMC1 and BOLL, whilst tubules expressing both markers were identified in adult testis tissue. Negative control (adult human testis). The images for ‘Continuous hCG’ and ‘Withdrawal hCG’ are representative of *n* = 27 and *n* = 4, respectively. yr: years. Scale bar: 20 μm.

Having determined the impact of hCG exposure on Leydig cell function and Sertoli cell development, we aimed to investigate its effect on germ cells. We characterized the germ cell populations in the human testis from childhood through to adulthood. In prepubertal testis tissues, spermatogonia expressing melanoma-associated antigen A4 (MAGE-A4^+^) were randomly distributed within the seminiferous cords (Figure 7A). The majority of MAGE-A4^+^ cells in the peripubertal testis were arranged at the basement membrane, and a similar distribution was observed in adult testis (Figure 7A). Meiotic cells could be identified during puberty (13-year-old) and in adulthood (Figure 6A).

We investigated whether germ cell survival and differentiation in grafts could be affected by exogenous hCG administration. Immature germ cell populations were identified by activated protein 2 gamma (AP-2γ; gonocytes) and (MAGE-A4; spermatogonia) and were found in all pre-graft human fetal testis tissues and all grafts taken from the ‘Continuous hCG’ and ‘Withdrawal hCG’ groups (Figure 7B). Germ cell proliferation was primarily restricted to the gonocyte subpopulation (AP2γ ^+^/Ki67^+^) in both the pre-graft controls and all (27/27) of the ‘Continuous hCG’-exposed grafts, whereas germ cell proliferation was not seen in any (0/4) of the ‘Withdrawal hCG’ grafts (Figure 7B and Figure S2). The initiation and progression of meiosis is required for the generation of spermatozoa from immature spermatogonia. Therefore, we investigated the expression of H2A histone family, member X (γH2AX), which marks cells with DNA double-strand breaks, including meiotic and apoptotic cells. As expected, γH2AX expression was observed in meiotic and post-meiotic cells in all adult human testes (Figure 7C). We also identified occasional γH2AX^+^ cells in pre-graft control testes and grafts from both treatment groups, which were negative for both apoptotic markers, cleaved-caspase 3 (c-Casp3) and cleaved-poly (ADP-ribose) polymerase (c-PARP) (Figure 7C). To determine whether γH2AX^+^ cells represented meioic cells, we examined the expression of two specific meiotic markers, DNA meiotic recombinase 1 (DMC1) and boule-like protein (BOLL). No DMC1 or BOLL positive cells were found in pre-graft controls or grafts, as opposed to adult testes in which both markers were detected (Figure 7D). Taken together, these results suggest that hCG stimulation does not induce the development of germ cells through meiosis in human testis xenografts.

## 4. Discussion

Sertoli cell maturation involves a series of structural and functional changes required to support germ cell differentiation into sperm in adulthood. Therefore, identifying the factors required for Sertoli cell maturation is crucial for the generation of sperm from cryopreserved immature human testis tissue as a fertility preservation strategy for adult survivors of childhood cancer.

In the present study, we therefore sought to investigate whether Sertoli cell maturation and Leydig cell steroidogenesis could be effectively induced by exposing human fetal testis to exogenous hCG in a xenotransplantation model, with a direct comparison to human testis tissues from fetal life, prepuberty, and adulthood. Crucially, we also characterised the normal progression of Sertoli cell development and Leydig cell function in the human testis throughout prepuberty and into adulthood.

The castration of host mice prevented the production of endogenous testosterone. Thus, seminal vesicle weights in the adult range for mice indicated active steroidogenesis and testosterone production from grafts exposed continuously to hCG. In grafts in which hCG was withdrawn, seminal vesicle weights were significantly smaller and steroidogenic cells (CYP11A1) were rarely identified, indicating that the xenografts were not producing testosterone.

The importance of Sertoli cell-specific AR signalling in germ cell differentiation has been demonstrated in knockout mouse models in which the loss of AR in Sertoli cells resulted in infertility [15,30,31]. Previous human fetal testis xenograft investigations showed either no expression [19] or the occasional (4% of cells) strong expression of AR in Sertoli cells in xenografts exposed to hCG for up to 20 weeks [22]. Conversely, we identified AR expression in the majority of Sertoli cells in continuously hCG-treated long-term xenografts. This is in agreement with an immature non-human primate testis xenotransplantation study, which detected AR in Sertoli cells in xenografts retrieved from gonadotrophin-exposed mice [23]. Taken together, our results indicate that continuous hCG exposure is necessary to maintain Leydig cell steroidogenesis and to induce androgen sensitivity (AR expression) in Sertoli cells.

At puberty, mature Sertoli cells form a unique microenvironment through the establishment of the BTB, which includes the expression of CX43 and CLDN11 at the basement membrane. Failure to build the blood–testis barrier during testis development is incompatible with complete germ cell differentiation, as evidenced by impaired spermatogenesis in CX43 and CLDN11 knockout mice [14,28]. Men with spermatogenic impairment also display an immature disorganized CX43 and CLDN11 spatial arrangement consistent with the loss of blood–testis barrier function [32]. The expression of CX43 within tubules of continuously hCG-treated grafts was strikingly similar to that observed in adult testis, suggesting a role of exogenous hCG stimulation in inducing an adult-like appearance of this gap junction protein, which was completely absent in the seminiferous cords of pre-graft control testes. In contrast to the findings for CX43, we did not observe changes towards an adult-like profile for CLDN11 in grafts for either treatment group, indicating that blood–testis barrier formation was incomplete.

Mature Sertoli cells begin to secrete seminiferous fluid during puberty, which results in the transformation of testis cords into seminiferous tubules possessing a lumen. We demonstrated lumen development in grafts exposed continuously to hCG, whilst no signs of lumen formation were observed in ‘Withdrawal hCG’ grafts. This is in accordance with a previous rodent study, which showed that testosterone pellets inserted under the tunica albuginea of 7-day-old rats induced the establishment of a lumen within the seminiferous tubules [33].

Terminally differentiated (pubertal/adult) Sertoli cells are identified as non-proliferative cells that no longer express markers detected in immature (fetal/prepubertal) Sertoli cells, including CK18 and AMH [29]. CK18 is recognized as an indicator of Sertoli cell immaturity and has been reported to exclusively be a fetal Sertoli cell marker [34], whilst Stosiek and colleagues showed that CK18 is also expressed postnatally in prepubertal testis [35]. In the present study, we show that CK18 is observed in fetal and prepubertal human testis with no expression detected by 5 years of age. We also found a loss of CK18 in grafts in both ‘Continuous hCG’ and ‘Withdrawal hCG’ groups, indicating that continuous hCG exposure is not required to downregulate its expression. In contrast, the cessation of Sertoli cell proliferation was dependent on continued hCG stimulation.

AMH downregulation is an important feature of Sertoli cell maturation. Although puberty is characterized by an inverse correlation between meiotic cells/testosterone levels and AMH expression, the mechanism for AMH downregulation in pubertal human testis has not been definitively shown. Fetal and early postnatal testes are exposed to testosterone; however, the lack of AMH downregulation during this period has been attributed to the absence of AR in immature Sertoli cells, which renders these cells unresponsive to testosterone stimulation [36]. In our study, despite the induction of AR expression in Sertoli cells and testosterone biosynthesis by Leydig cells, AMH expression was detected in grafts exposed continuously to hCG, suggesting that testosterone acting via AR might not be sufficient to fully downregulate AMH expression. These results are in keeping with earlier work in mice, which showed that when AR in Sertoli cells was knocked out, AMH downregulation occurred at the same time as it did in the control [31]. Further evidence for AR-independent AMH downregulation has been demonstrated in a gain of function transgenic Sertoli cell-specific AR (TgSCAR) mouse model [37].

Previous investigations involving the xenotransplantaion of prepubertal human testis tissue have mainly focused on evaluating the status of germ cell differentiation in xenografts, and the characterization of the somatic cell populations (e.g., Sertoli cell maturation, Leydig cell steroidogenesis) has been less explored. These studies have reported the survival of spermatogonia similar to the present study [38,39,40] or the initiation (but not progression) of meiosis [41,42,43]. Although germ cells survived in grafts, progression through meiosis did not occur in our study. This may be related to the fact that Sertoli cells in continuously hCG-treated grafts were not terminally differentiated, as indicated by persistent AMH expression and an immature CLDN11 distribution profile. Given the limited availabilty of the immature human testis tissue allocated to research, the mechanisms that may underlie gonadotrophin effects on testicular development have not been explored in this study and require further investigation. The absence of germ cell differentiation in our study can potentially be overcome by grafting for a longer period. Given that the lifespan of nude mice is ∼2 years, the retrieval of grafts followed by re-grafting into younger recipients might also be an option. Moreover, the (i) identification of additional factors that induce complete maturation of Sertoli cells, (ii) the use of regimens that more closely resemble the sequential hormonal profile of the infantile, prepubertal, and pubertal period, and (iii) the investigation of the synergic action of hCG and follicle-stimulating hormone (FSH) may potentially induce somatic and germ cell differentiation. In clinical practice, hCG monotherapy, combined hCG and FSH therapy, and sequential gonadotrophin therapy (FSH pre-treatment followed by hCG and FSH) are considered as effective fertility-inducing treatments for gonadotrophin-deficient men [44,45,46].

## 5. Conclusions

In conclusion, we demonstrate, in a xenotransplantation model, the potential for hCG to induce Sertoli cell maturation and to maintain the functionality of Leydig cells. We provide evidence for the acquisition of many features observed in mature Sertoli cells that are associated with androgen responsiveness, including the expression of AR, CX43, and lumen development. However, Sertoli cells retained the expression of AMH, indicating incomplete maturation and supporting the hypothesis that AMH downregulation might be independant of androgen stimulation. Moreover, we provide, for the first time, the spatiotemporal expression of key components of the BTB (CX43 and CLDN11) across testis development (from fetal life to adulthood).

Terminally differentiated Sertoli cells contribute to germ and Leydig cell differentiation and blood–testis barrier development. They also have the ability to modulate the immune response through the production of immune regulatory factors. This means that Sertoli cells may also be useful in a variety of clinical applications, such as the use of encapsulated Sertoli cells to deliver immune modulatory and trophic factors [47,48,49,50], as a cell-based drug delivery system [51], or being engineered to express therapeutic proteins [52,53,54], which may be used to treat cancer, chronic, and autoimmune diseases. Therefore, understanding the factors involved in human Sertoli cell maturation may also have relevance to these developing fields of clinical research.

Data presented in this study expand our understanding of factors implicated in the process of human Sertoli cell maturation and may be of direct relevance to future investigations aimed at developing fertility preservation strategies for prepubertal boys due to undergo gonadotoxic treatments. 

## Figures and Tables

**Table 1:** Antibodies used for immunofluorescence.

Antibody	Cell Type	Supplier	Cat. No.	Dilution
CK18 (DC-10)	Sertoli cell (immature)	Santa Cruz Biotechnology	sc-6259	1:200
MIS (C-20) (AMH)	Sertoli cell (immature)	Santa Cruz Biotechnology	sc-6886	1:1000
AR (N-20)	Sertoli cell * (mature)	Santa Cruz Biotechnology	sc-816	1:2000
SOX9	Sertoli cell (all stages)	Millipore	AB5535	1:10,000
CX43	Sertoli cell (BTB)	Cell Signaling Technology	3512	1:300
CLDN11	Sertoli cell (BTB)	Thermo Fisher Scientific	36-4500	1:500
CYP11A1	Leydig cell (steroidogenesis)	Sigma-Aldrich	HPA016436	1:5000
AP2γ	Germ cell (Gonocyte)	Santa Cruz Biotechnology	sc-12762	1:75
MAGE-A4	Germ cell (Spermatogonia)	Gift ^#^	-	1:200
γH2AX	Germ cell ** (meiotic)	Abcam	AB26350	1:1000
DMC1	Germ cell (meiotic)	Abcam	AB11054	1:4000
BOLL	Germ cell (meiotic)	Novus Bio	H00066037-M03	1:1000
Ki67	Proliferating cell	Abcam	AB16667	1:100
c-PARP	Apoptotic cell	Cell Signaling Technology	5625	1:100
c-Caspase-3	Apoptotic cell	Cell Signaling Technology	9661	1:100
Hoechst	DNA counterstain	Thermo Fisher Scientific	33342	1:4000

* Also expressed in Leydig- and peritubular myoid cells. ** Also expressed in apoptotic cells. ^#^ Gift from Professor Giulio Spagnoli (University of Basel).

## Supplementary Materials

The following are available online at https://www.mdpi.com/2077-0383/9/1/266/s1.
