# Supplementary material for: Exogenous Gonadotrophin Stimulation Induces Partial Maturation of Human Sertoli Cells in a Testicular Xenotransplantation Model for Fertility Preservation
Source: J Clin Med. 2020 Jan 18;9(1):266. doi: 10.3390/jcm9010266 (PMC7019512; doi:10.3390/jcm9010266)
Supplement: Supplementary file 1 [file jcm-09-00266-s001.pdf]

Supplementary Material

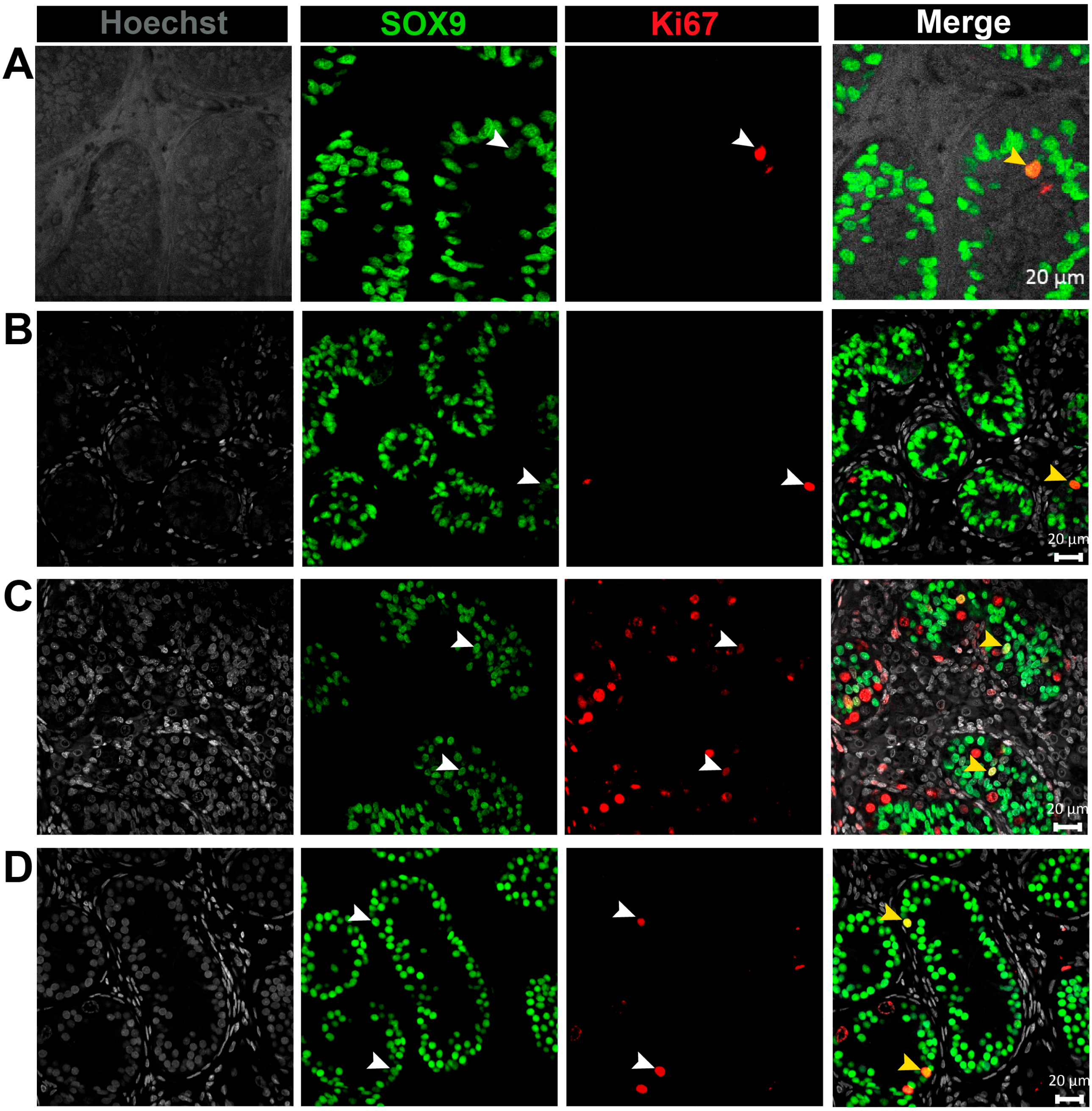

Figure S1. Co-localisation of SOX9 and Ki67 expression in immature testis tissue. **(A**) Ungrafted testis tissue from 1-year-old patient. (**B)** Ungrafted testis tissue from 2-year-old patient. **(C)** Pre-graft control. **(D)** Graft recovered from mice exposed to hCG for 7 months followed by 5 months withdrawal hCG. Yellow arrowheads indicate SOX9$^+$/Ki67$^+$ cells (proliferating Sertoli cells). White arrows indicate corresponding cells in single channel images. Scale bar: 20 µm.

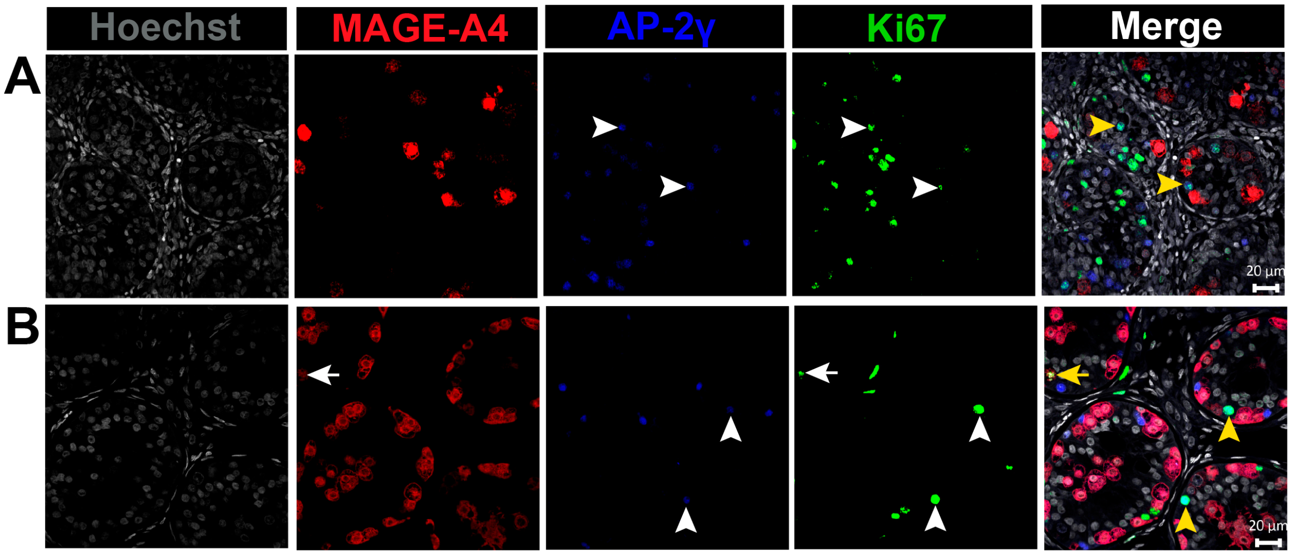

Figure S2. Co-localisation of MAGE-A4, AP2γ and Ki67 expression in immature testis tissue. **(A)** Pre-graft control. **(B)** Long-term xenograft exposed continuously to hCG. Yellow arrowheads denote AP2γ⁺/Ki67⁺ cells (proliferating gonocytes). Yellow arrow indicates MAGE-A4⁺/Ki67⁺ cells (proliferating spermatogonia). White arrows and arrowheads indicate corresponding cells in single channel images. Scale bar: 20 µm
